# Exhaled Breath Condensate as a Suitable Matrix to Assess Lung Dose and Effects in Workers Exposed to Cobalt and Tungsten

**DOI:** 10.1289/ehp.7108

**Published:** 2004-06-10

**Authors:** Matteo Goldoni, Simona Catalani, Giuseppe De Palma, Paola Manini, Olga Acampa, Massimo Corradi, Roberto Bergonzi, Pietro Apostoli, Antonio Mutti

**Affiliations:** ^1^National Institute of Occupational Safety and Prevention, Research Centre at the University of Parma, Parma, Italy; ^2^Department of Clinical Medicine, Nephrology and Health Sciences, University of Parma, Parma, Italy; ^3^Department of Experimental and Applied Medicine, University of Brescia, Brescia, Italy

**Keywords:** cobalt, exhaled breath condensate, hard metals, lung, malondialdehyde, oxidative stress, tungsten

## Abstract

The aim of the present study was to investigate whether exhaled breath condensate (EBC), a fluid formed by cooling exhaled air, can be used as a suitable matrix to assess target tissue dose and effects of inhaled cobalt and tungsten, using EBC malondialdehyde (MDA) as a biomarker of pulmonary oxidative stress. Thirty-three workers exposed to Co and W in workshops producing either diamond tools or hard-metal mechanical parts participated in this study. Two EBC and urinary samples were collected: one before and one at the end of the work shift. Controls were selected among nonexposed workers. Co, W, and MDA in EBC were analyzed with analytical methods based on mass spectrometric reference techniques. In the EBC from controls, Co was detectable at ultratrace levels, whereas W was undetectable. In exposed workers, EBC Co ranged from a few to several hundred nanomoles per liter. Corresponding W levels ranged from undetectable to several tens of nanomoles per liter. A parallel trend was observed for much higher urinary levels. Both Co and W in biological media were higher at the end of the work shift in comparison with preexposure values. In EBC, MDA levels were increased depending on Co concentration and were enhanced by coexposure to W. Such a correlation between EBC MDA and both Co and W levels was not observed with urinary concentration of either element. These results suggest the potential usefulness of EBC to complete and integrate biomonitoring and health surveillance procedures among workers exposed to mixtures of transition elements and hard metals.

Cobalt is a transition element occurring in four valences (0, +2, +3, and +4), the divalent oxidation state being the most common. Tungsten, also known as wolfram (W), is a hard metal that can occur in the natural state only in the form of chemical compounds with other elements. Co is a major constituent (5–10%) of hard metal alloys, mainly based (> 80%) on tungsten carbide (W-C) and small percentages of other carbides (Lauwerys and Lison 1994). Other industrial uses of Co include diamond polishing with Co-containing disks and production of drying agents, pigments, and catalysts. W and its alloys are used extensively for filaments for electric lamps, electron and television tubes, and metal evaporation work. W has no recognized physiologic roles, whereas Co is a cofactor of vitamin B_12_ and therefore is an essential element (Taylor and Marks 1978).

Occupational exposure to Co can lead to various lung diseases, such as interstitial pneumonitis, fibrosis, and asthma [Agency for Toxic Substances and Disease Registry (ATSDR) 1999; Cirla 1994; Lauwerys and Lison 1994; Lison 1996; Nordberg 1994]. Although the mechanisms of Co-induced lung toxicity are not completely known, there is evidence from both *in vivo* and *in vitro* experiments supporting the view that Co induces the production of reactive oxygen species (ROS) with subsequent oxidative stress [reviewed by Lison et al. (2001); Nemery et al. (1994); Salnikow et al. (2000)]. In addition, ROS generation by Co administration is significantly increased by coexposure to W-C particles, through a physical–chemical mechanism of interaction (Lison et al. 1995). Although W-C alone seems to cause marginal genotoxic effects (De Boeck et al. 2003; Van Goethem et al. 1997), when administered alone it would be unable to induce lung parenchymal lesions (Lasfargues et al. 1992) and oxidative stress (Lison et al. 1995). Although Co was classified as group 2B, possibly carcinogenic to humans [International Agency for Research on Cancer (IARC) 1991], its association with W-C has recently been included in group 2A, probably carcinogenic to humans (IARC 2003). Therefore, strict control of dust level and regular health monitoring are recommended for workers employed in the hard metal industry, where such a coexposure may occur.

Urinary Co excretion (Co-U) has been proposed as a biomarker of exposure because of its correlation with airborne Co concentration (Alexandersson 1988; Apostoli et al. 1994; Christensen and Poulsen 1994; Linnainmaa and Kiilunen 1997; Scansetti et al. 1985). Little information is available about W metabolism and kinetics, mainly because of the lack of suitable methods for its determination in biological matrices other than the relatively recent and expensive technique based on inductively coupled plasma–mass spectrometry (ICP-MS). W measurement in urine has been proposed as a suitable exposure biomarker in the hard metal industry, where exposure to W-C is variable and has been reported to reach 417 μg/m^3^ (Kraus et al. 2001).

A key issue for risk assessment in occupational health is the characterization of dose at the target organ level. In the case of inhalable pneumotoxic metals, such as Co and W, a noninvasive method for sampling from the lung would be extremely useful. Standard methods for the evaluation of lung pathobiology (bronchoscopy, induced sputum) have a high degree of invasiveness, which limits their applicability to occupational monitoring. Exhaled breath condensate (EBC), obtained by cooling exhaled air under condition of spontaneous breathing, is a new technique to assess pulmonary status, and published data suggest that its composition has a good correlation with bronchoscopic specimens (Kharitonov and Barnes 2001; Mutlu et al. 2001). EBC is a noninvasive and simple procedure, and portable devices have been developed; therefore, it has the potential for application in occupational settings (Antczak and Gorski 2002; Lemiere 2002). Preliminary studies on smokers and patients with chronic obstructive pulmonary disease have revealed that several toxic metals and trace elements are detectable in EBC, raising the possibility of using this new matrix to quantify the lung tissue dose of pneumotoxic substances (Mutti et al. 2003). Furthermore, EBC analysis can be used to quantify relevant biomarkers reflecting lipid peroxidation of membrane cells (Corradi et al. 2003; Kharitonov and Barnes 2001; Mutlu et al. 2001). To the best of our knowledge, EBC analysis has never been used in occupational settings.

The aim of the present study was to investigate whether EBC can be employed for a better risk assessment among workers exposed to pneumotoxic metals, using workers from the hard metal industry as a first paradigmatic application.

## Materials and Methods

### Subjects.

Thirty-three workers from three factories producing either diamond tools (group A, *n* = 12 subjects; group B, *n* = 11 subjects) or hard-metal inserts (group C, *n* = 10 subjects) were recruited to participate to this study. Groups A and B were exposed either to Co and W-C powders, which were mixed to produce hard-metal alloys by synterization, or to metal dust originating from dry-grinding activities. Subjects from group C were exposed to metal dust produced during the dry grinding of hard-metal pieces. Sixteen adult healthy subjects, not occupationally exposed to metals, were recruited as the control group. Controls were defined as individuals with normal spirometry and without a significant history of lung diseases.

Demographic and clinical data of participants are summarized in Table 1. Workers belonging to groups A, B, and C presented normal spirometric indexes, with the exception of a single worker (heavy smoker) from group B showing mild airway obstruction. Two subjects from group A and one from group C had a history of mild intermittent asthma, and both were under therapy with inhaled salbutamol as needed. One subject from group B had radiologic evidence of pulmonary fibrosis ascribed to the occupational exposure to hard metals. All other workers were asymptomatic and did not refer a significant current or past respiratory diseases. Symptoms of acute respiratory illness within the 4 weeks preceding the study were ruled out in all subjects. All workers participating in the study denied vitamin B_12_ supplementation or beer drinking (which are potential confounders as sources of Co) over the week preceding the study.

### Study design.

Workers belonging to groups A and C were evaluated both before (~ 15 hr after the end of the last exposure) and at the end of the same 8-hr work shift. Samples from subjects in group B were obtained only at the end of the work shift. In addition to EBC and urine collection, a short questionnaire about current and past medical history was completed and a spirometry was performed. We carried out the same procedures in controls in our laboratory during a normal working day. In order to verify whether possible air contamination in the offices could influence exhaled metals, three controls collected EBC inside the offices of the plants. Because the levels of Co and W in EBC of these subjects were undetectable, we concluded that the contamination of office environmental air, if any, provided a negligible contribution to the concentration of these elements in EBC.

All subjects enrolled in the study provided written informed consent to the procedures, which were approved by the local institutional human ethical committee. The sampling of biological material was carried out according to the Declaration of Helsinki (World Medical Association 2002).

### Spirometric measurements.

Spirometry was performed with a pneumotachograph (Koko Spirometer; Sensormedics, Milan, Italy). We obtained mean values for forced expiratory volume in the first second (FEV_1_) and forced vital capacity (FVC) from the three best acceptable test values of lung function, according to the recommendation of the American Thoracic Society (1995).

### Environmental measurements.

Environmental monitoring was carried out by stationary samplers. The inhalable fraction of particulate matter was collected using a selector, following the procedures suggested by the American Conference of Governmental Industrial Hygienists (ACGIH 2003). Airborne particulate was collected on cellulose ester membranes (0.8 μm porosity, 25 mm diameter) at a constant flow of 3 L/min for a period ranging from 4 to 7 hr during the same day of biological monitoring. Membranes conditioned before and after dust sampling were weighted in a thermohygrometrically conditioned cabinet using a precision microbalance reading 0.0001 mg. Membranes were then dissolved in concentrated hyperpure nitric acid, and the solution was diluted with ultrapure water. The analytical blank was obtained from virgin membranes, nitric acid, and water. Co and W were analyzed by an ICP-MS instrument using the same method applied to analyze biological samples (Apostoli et al. 1998). Measured dusts were expressed as micrograms per cubic meter.

Airborne concentrations of Co and W are reported in Table 2. The highest concentrations of Co and W were observed in factory 3 (group C). In factories 1 and 2 (groups A and B, respectively), comparable Co levels were observed, whereas factory 2 showed slightly higher W levels.

We did not use personnel biomonitoring of Co and W, mainly because, in the three plants considered, the work places were very narrow and the workers had almost no mobility within the working area. Therefore, the collectors were placed in the proximity of the workers’ breathing area. In addition, because of the expected low airborne levels, the use of stationary samplers allowed the use of higher flow rates, thereby increasing sampling efficiency and reducing analytical errors, which may have a greater impact at relatively low airborne concentrations.

### EBC collection.

EBC was collected with a simple homemade apparatus formed by five components: *a*) a mouthpiece set up to work also as a saliva trap; *b*) a nonrebreathing polypropylene valve; *c*) a 10-cm Tygon tube (Nalgene 890 FEP tubing; Nalge Nunc International, Rochester, New York, USA); *d*) a 50-mL polypropylene vial; and *e*) a Dewar flask refrigerated with gel refrigerant (Ice-Brix; BDH Laboratory Supplies, Poole, Dorset, UK); the apparatus was placed at –20°C the night before the measurements. The five disposable components can be easily assembled, giving rise to simple portable apparatus essentially composed of two parts: *a*) a disposable part, which is maintained at room temperature and is composed of the mouthpiece with the rebreathing valve and the tube (which connects the valve to the vial); and *b*) the condensing part, which is composed of a disposable vial immersed in the refrigerant gel inside the Dewar flask. Inside the Dewar flask kept at room temperature, the gel refrigerant remains completely frozen up to 6 hr at a constant temperature (–20°C). Therefore, it can be used for several EBC collections. Exhaled air condenses along the internal surface of the vial, whose temperature is close to –20°C. Upon condensation, EBC droplets collect at the bottom of the tube, which, unlike the tube walls, is not in contact with the refrigerant gel; this keeps the temperature slightly above 0°C. Therefore, EBC collected with this device remains liquid. This is an important feature because some proteins and peptides may change their quaternary structure and lose immunoreactivity upon repeated thawing and freezing.

In EBC samples collected with this device from 12 subjects, salivary contamination was excluded through the colorimetric detection of α-amylase (Infinity amylase reagent; Sigma, St. Louis, MO, USA). Any possible release of Co and W from plastics or contamination during EBC collection was excluded in repeated experiments made by extensive washing of each component of the collection circuit (data not shown).

Subjects were asked to breathe tidally through the mouthpiece for 10 min, sitting comfortably in the workplace office (workers) or in our laboratory (controls). Subjects were instructed to form a complete seal around the mouthpiece with their mouth and to maintain a dry mouth during collection by periodically swallowing excess saliva. In addition, they were asked to rinse their mouths thoroughly before the maneuver and each 5 min during the test. To prevent any contamination from skin, subjects were asked to wash their hands before EBC and urine collection and to wear disposable latex gloves during the collecting procedures. EBC samples (almost 1 mL) were transported in dry ice to the laboratory and stored at –80°C in polypropylene tubes until analytical determinations.

### Analyses of Co and W in urine and EBC.

Co-U and urinary excretions of W (W-U) were measured using flow injection (FI) ICP-MS and expressed as a function of creatinine, as previously described (Apostoli et al. 1998).

The protocol used for water analysis was applied to EBC samples because EBC does not show any matrix effect. Briefly, 2.5 mL of ultrapure bidistilled water (MilliQ; Millipore, Milan, Italy) was added to 0.5 mL EBC, which was then vigorously shaken before analysis by FI ICP-MS. We determined the accuracy of methods by means of analyzing standard reference material 1640 [National Institute of Standards and Technology (NIST) Gaithersburg, MD, USA]. The precision expressed as coefficient of variation varied from 4 to 8% among series and from 6 to 12% between the series. The detection limit, determined on the basis of 3 SDs of the background signal, was 0.003 μg/L for both Co and W.

**Figure 1:**
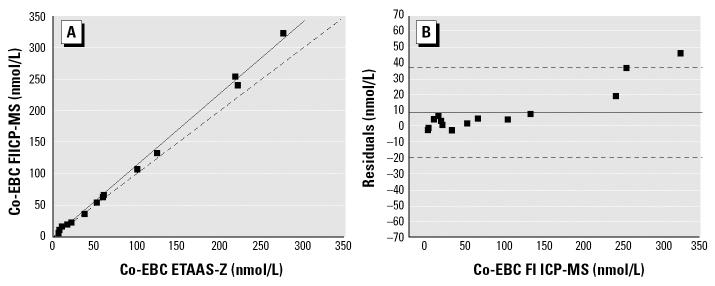
Correlation (*A*) and Bland-Altman graph (*B*) of the comparison between ETAAS-Z and FI ICP-MS techniques for the measurements in 12 EBC samples. In (A), the solid line represents the best fit of experimental values, and the dashed line shows the theoretical identity line. In (*B*), the solid line shows the mean deviation between the two methods; dashed lines indicate the mean ± 2 SD. *A* = –3.08381 ± 2.28647; *B* = 1.14266 ± 0.01921; *R* = 0.99817; *p* < 0.0001.

The method for Co analysis in EBC was compared with the most used technique relying on atomic absorption spectroscopy with Zeeman background correction (ETAAS-Z). The agreement between the two methods was assessed both by correlation analysis and by applying the Bland-Altman method, a suitable approach to verify the agreement between two independent methods (Bland and Altman 1986), and is presented in Figure 1.

### Determination of malondialdehyde (MDA) in EBC.

We determined MDA in EBC (MDA-EBC) by liquid chromatography tandem mass spectrometry (LC-MS/MS) using an Applied Biosystems-Sciex API 365 triple quadrupole mass spectrometer (Sciex, Concord, Ontario, Canada) equipped with a heated nebulized interface for atmospheric pressure chemical ionization. Aldehydes were separated by reversed-phase liquid chromatography after derivatization with dinitrophenylhydrazine, as previously described (Andreoli et al. 2003). The limit of detection was 1.0 nmol/L of MDA, and the limit of quantitation was 3 nmol/L.

### Statistical analyses.

Statistical analysis was performed using SPSS 11.5 software (SPSS, Chicago, IL, USA). When separate groups were considered, Co and W in biological media were not normally distributed. Also, the corresponding log-transformed values did not result in Gaussian distributions (one-sample Kolmogorov-Smirnov *Z* and Shapiro-Wilk test). Therefore, results were reported as median and range, and Wilcoxon and Mann-Whitney tests were used to assess differences between groups. Owing to the lack of interference of tobacco smoking on metal levels, further statistical analyses were performed irrespective of smoking habits.

In the overall sample, all measured parameters showed a log-normal distribution (Kolmogorov-Smirnov *Z* and Shapiro-Wilk test). Therefore, all regression analyses were performed on log-transformed values. Pearson’s *R* was used to assess the correlation between variables, with *p*-values < 0.05 (two-tailed) considered statistically significant. To compare the difference between the slopes of different regression lines, we used an appropriate form of Student’s *t*-test (Glantz 2002). Finally, to assess the effect of Co and W on the MDA levels, we performed an analysis of covariance (ANCOVA) on log-transformed values. The significance level for all the used tests was *p* = 0.05.

## Results

Analytical determinations on urine and EBC samples collected at the end of the work shift are summarized in Table 3. In controls, Co-EBC was measurable by ICP-MS but not by ETAAS-Z, whereas both techniques revealed measurable and much higher levels in all exposed workers. We observed a similar behavior for Co-U, whose values among exposed workers were higher by several orders of magnitude compared with both controls and the corresponding Co-EBC concentrations.

In samples collected before the work shift among workers from group A, neither Co-EBC [median, 2.0 nmol/L (interquartile range, 0.5–3.4 nmol/L)] nor Co-U [2.1 μmol/mol creatinine (1.7–3.8 μmol/mol)] was significantly different compared with controls. In group C, basal levels of both Co-EBC [median, 57.7 nmol/L creatinine (interquartile range, 10.2–219.0 μmol/mol creatinine)] and Co-U [7.8 μmol/mol creatinine (2.7–11.8 μmol/mol creatinine)] were much higher than control values (*p* < 0.01).

In workers from groups A and C, paired analysis showed significant increases in Co-EBC levels at the end of exposure compared with values recorded before the working shift (*p* < 0.01 and *p* < 0.05, respectively). In workers from group C, but not from group A, a significant difference between preexposure and postexposure levels was observed for Co-U (*p* < 0.01).

At both sampling times, W was not detectable in EBC or in most urine samples from group A workers and controls. In samples collected from group C workers before the working shift, W-EBC [median, 8.7 nmol/L (interquartile range, 3.3–16.9)] and W-U [2.3 μmol/mol creatinine (1.1–3.5 μmol/mol creatinine)] were higher than were control values but about three times lower compared with the corresponding postexposure levels reported in Table 3 (*p* < 0.01).

MDA-EBC was measurable in all samples. In groups B and C, postexposure MDA-EBC levels were higher than control values (*p* < 0.01 and *p* < 0.05, respectively), whereas the difference did not reach statistical significance for less-exposed workers in group A (*p* = 0.1). A significant increase in MDA-EBC values over preexposure levels [median, 9.2 nmol/L (interquartile range, 5.2–15.0 nmol/L )] was apparent in group C (*p* < 0.01), whereas no differences in MDA-EBC levels were observed in group A compared with preexposure concentrations [7.9 nmol/L (6.5–10.3 nmol/L)].

Co-EBC levels did not correlate with airborne Co (*R* = 0.27, *p* = 0.15). A positive correlation between airborne Co and Co-U levels was observed in groups A and C, either considered separately (*R* = 0.77, *p* < 0.01, and *R* = 0.78, *p* < 0.01, respectively) or taken together (*R* = 0.79, *p* < 0.01). The inclusion of group B lowered the correlation coefficient (*R* = 0.46, *p* < 0.01). In group B, the correlation between airborne Co and Co-U levels observed in groups A and C was no longer apparent. In workers from group C only, a positive correlation (*R* = 0.70, *p* < 0.05) was observed between airborne W and W-U levels.

In group B, we observed an apparent inconsistency between the relatively low airborne Co levels and the high concentrations of Co-U. A possible explanation is represented by poor hygienic conditions (a couple of workers smoked during work hours), which may lead to hand contamination and Co absorption from routes other than inhalation (dermal and oral).

**Figure 2:**
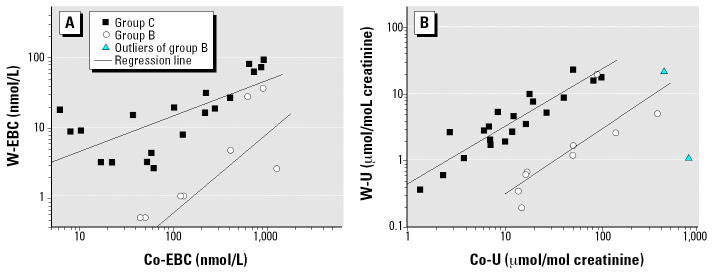
Correlation (*A*) between Co-EBC and W-EBC levels and (*B*) between Co-U and W-U levels. In (*A*), group B log(W-EBC) = 2.44 + log(Co-EBC^1.12^)and Group C log(W-EBC) = 0.15 + log(Co-EBC^0.51^). In (*B*), group B log(W-U) = 1.47 + log(Co-EBC^0.97^) and Group C log(W-U = 0.35 + log(Co-EBC^0.86^).

In preexposure and postexposure pooled samples (Figure 2A), Co-EBC was significantly related to W-EBC both for group B (*R* = 0.70, *p* < 0.05) and group C (*R* = 0.72, *p* < 0.01), although with a different slope (1.11 ± 0.41 for group B vs. 0.50 ± 0.11 for group C, mean ± SEM). In groups B and C, a similar correlation was also found (Figure 2B) between Co-U and W-U (*R* = 0.80, *p* < 0.01, and *R* = 0.91, *p* < 0.01, respectively). Although we observed no difference between the slopes of the two regression lines (0.97 ± 0.27 for group B vs. 0.86 ± 0.10 for group C), a shift between the intercepts of the two regression lines was evident.

**Figure 3:**
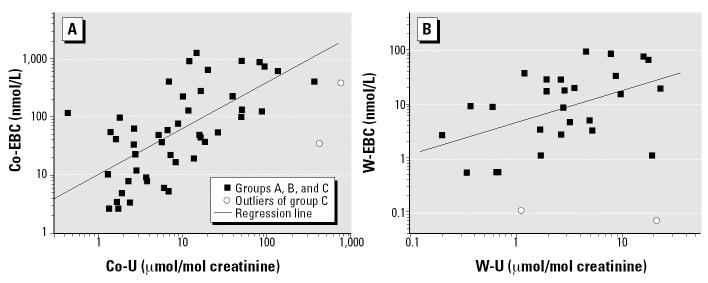
Correlation between (*A*) Co-U and Co-EBC levels and (*B*) between W-U and W-EBC levels. In (*A*), log(Co-EBC) = 0.99 + log(Co-U^0.79^); in (*B*), log(W-EBC) = 0.65 + log(W-U^0.59^).

In a pooled analysis of preexposure and postexposure samples from all workers (Figure 3A), Co-EBC correlated with Co-U (*R* = 0.62, *p* < 0.01). A similar relationship on pooled data (Figure 3B) was observed between W-U and W-EBC (*R* = 0.48, *p* < 0.01).

In group C, MDA-EBC levels at both times were significantly correlated with Co-EBC (*R* = 0.91, *p* < 0.01) but not with Co-U. In workers from groups A and B, such a correlation was apparent in postexposure samples (*R* = 0.68, *p* < 0.05, and *R* = 0.67, *p* < 0.05, respectively). In group C, we also found a correlation between W-EBC and MDA-EBC (*R* = 0.77, *p* < 0.01).

**Figure 4:**
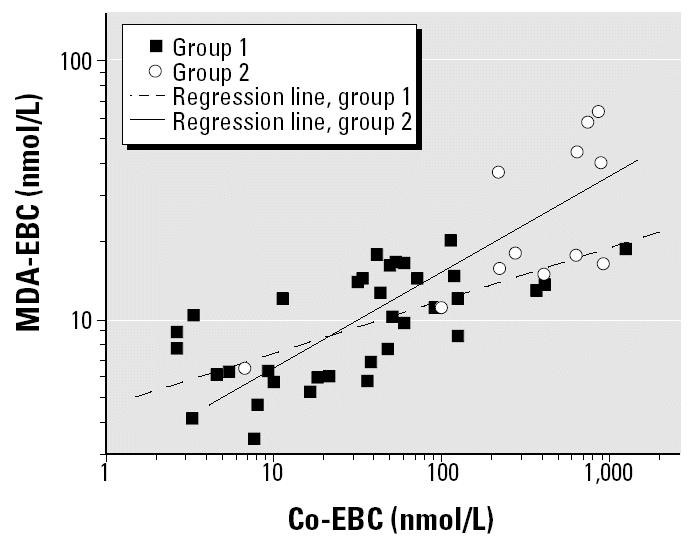
Correlation between Co-EBC and MDA-EBC levels. Group 1, workers with W-EBC < 16.3 nmol/L [log(MDA-EBC) = 0.66 + log(Co-EBC^0.21^)]; group 2, workers with W-EBC > 16.3 nmol/L [log(MDA-EBC) = 0.44 + log(Co-EBC^0.37^)].

To test whether the concomitant W exposure interferes with Co pneumotoxicity, we identified two different groups of workers on the basis of the median of W-EBC. Regression lines between Co-EBC and MDA-EBC among subjects with W-EBC, respectively, lower and higher than 16.3 nmol/L (Figure 4) showed different slopes (mean ± SEM, 0.21 ± 0.04 vs. 0.37 ± 0.11; *p* < 0.01, Student’s *t*-test for the regression lines). In an ANCOVA model using MDA-EBC as the dependent variable, both W-EBC (*p* = 0.002) and Co-EBC (*p* < 0.001) had a significant influence on the variability of MDA-EBC. A highly significant Co by W interaction was also observed (*p* < 0.001). Interestingly, smoking habits did not enter in the model either alone or in combination with covariates.

## Discussion

The present study demonstrates that Co and W can be measured in the EBC of occupationally exposed workers and thus suggests the potential use of this matrix as a novel approach to monitor target tissue dose and effects occurring in the respiratory tract upon exposure to pneumotoxic substances. Indeed, inhaled toxic chemicals can act locally on the lung, which represents the route of entry of most environmental pollutants.

Biological monitoring of exposure to trace elements and toxic metals is mainly based on their measurement in blood and urine. However, these biomarkers integrate the overall intake from different absorption routes and can be used to assess the risk of systemic effects, rather than local effects on the respiratory tract. These limitations are confirmed in the present study by the lack of correlation between either Co-U or W-U and MDA concentration in EBC. On the contrary, both Co and W in EBC were correlated with MDA-EBC levels, thus suggesting that exhaled elements may reflect the lung dose responsible for local toxic effects. In addition, the relationship between Co levels in EBC and urine seems to indicate that Co-EBC really may represent the fraction of body dose (represented by Co-U), which has been inhaled and has not yet moved from lung tissue in the systemic circulation at the sampling time. However, the relatively weak (for W in particular) correlation between Co and W levels in EBC and the respective urinary levels suggests that other absorption routes (i.e., dermal and oral absorption) may provide a sizable contribution to absorbed dose and subsequent urinary excretion. Furthermore, the different bioavailability and solubility of Co and W (Kraus et al. 2001) could account for the weak correlation between the levels into two compartments.

Because of the novelty of the EBC measurements, the analytical validity of determinations was strictly controlled. We employed analytical methods based on mass spectrometric reference techniques, namely, LC-MS/MS for MDA and FI ICP-MS for metals, in order to obtain accurate and reliable results. Compared with other biological matrices, such as urine and blood, EBC represents a “clean” matrix, consisting mostly of water. The reliability of the elemental measurements in EBC was also assessed by performing the analysis of Co in EBC samples with two techniques relying on different principles, namely, FI ICP-MS and ETAAS-Z. The results showed a high repeatability, and therefore Co in EBC can be easily measured relying on a readily available technique such as ETAAS-Z.

The assessment of metal concentrations in different biological fluids can also provide useful information about kinetics and a better comprehension of physical–chemical interactions between metals, for example, between Co and W. In the present study, Co-U was related to both air concentrations and Co-EBC, despite the lack of correlation between Co-EBC and airborne Co. This could be consistent with a fast kinetic of inhaled Co, which can be readily absorbed via the lungs in the organism (ATSDR 1999). Therefore, we speculate that Co-EBC reflects not only exposure but also the amount of the element retained in the lung and eliminated with exhaled air after its interaction with—and possibly damage to—resident cells. In fact, there is a relationship between MDA-EBC (a marker of lipoperoxidation of membranes) and Co-EBC, which might represent a marker of effective dose or dose at the target. The correlation between MDA-EBC and Co-EBC is consistent with the mechanism of action of Co, which is known to cause oxidative stress (Lewis et al. 1991; Lison et al. 1995, 2001; Nemery et al. 1994). Considering that Co-U decreases rapidly (within 24 hr) after Co exposure has ceased (Alexandersson 1988; Apostoli et al. 1994), it is likely that the kinetics of absorption of Co at the level of the primary target organ (respiratory tract) are even faster. This is also consistent with the drastic drop in Co-EBC values at preexposure level about 16 hr after the last exposure.

A better understanding of the physical–chemical interactions between Co and W *in vivo* is another issue to which EBC can contribute. Exposure to W was quite different despite a similar productive cycle, probably depending on a different composition of hard metal alloys (covered by industrial secrets) and, perhaps, on different behaviors of workers from the point of view of personal hygiene. In a couple of outliers, urinary levels were higher by orders of magnitude than expected on the basis of the corresponding air and EBC concentrations. The significant Co-EBC by W-EBC interaction in the ANCOVA model with MDA-EBC as a dependent variable strongly suggests that W exposure has a synergistic effect *in vivo* on Co pneumotoxicity. This is in agreement with published data obtained from *in vitro* experiments (Lison et al. 2001). In fact, although W-C alone is known to be inert, there is some evidence that the physical–chemical association of Co and W-C generates electrons (provided by Co and transferred on the surface of W-C), which can reduce oxygen, thus giving rise to ROS (Lison et al. 1995).

Although Figure 2 shows that the levels of Co and W in EBC and urine are factory dependent, the concentration of MDA in EBC is strongly associated with both Co and W levels in EBC, thus confirming the most recent understanding of hard metal lung disease as a consequence of the combined effects of these elements.

In conclusion, the present study shows that EBC analysis is a promising matrix to assess the target tissue dose of pneumotoxic substances from polluted workplaces and to assess early effect markers, such as MDA.

## Figures and Tables

**Table 1:** Demographic and clinical characteristics of study groups.

Characteristic	Controls	Group A	Group B	Group C
No. of subjects	16	10	11	12
Age (years)	34.8 ± 2.1	39.1 ± 3.9	33.2 ± 2.0	37.9 ± 3.2
Sex (male/female)	11/5	10/0	10/1	8/4
No. of current/ex-/never-smokers	6/1/9	4/5/1	8/0/3	6/0/6
Pack-years of current/ex-smokers (mean ± SEM)	10.2 ± 1.5/20	13.1 ± 5.9/10.2 ± 2.9	12.6 ± 4.5/0	7.8 ± 1.5/0
FVC, percent of predicted (mean ± SEM)	104.3 ± 4.3	114.7 ± 11.0	98.5 ± 2.8	117 ± 3.4
FEV_1_, percent of predicted (mean ± SEM)	102.5 ± 0.1	116.5 ± 8.4	96.3 ± 3.4	110 ± 3.5
FVC/FEV_1_, percent (mean ± SEM)	83.1 ± 0.01	84.5 ± 1.0	81.9 ± 2.09	98 ± 1.5

**Table 2:** Airborne concentrations (mg/m^3^) of Co and W [median (range)] measured in the three working environments.

Group	Co	W
A	8.25 (0.1–16.4)	< 0.01
B	8.45 (0.9–16.0)	0.10 (0.01–0.2)
C	26 (14.6–37.4)	3 (1.1–4.9)

**Table 3:** End-of-shift values [median (interquartile range)] of biomarkers in the three factories.

Variables	Controls (*n* = 16)	Group A (*n* = 10)	Group B (*n* = 11)	Group C (*n* = 12)
Co-EBC (nmol/L)	0.7 (0.5–1.0)	40.7 (11.9–54.3)**	126 (44.1–628)**	163 (37.3–741)**
Co-U (μmol/mol creatinine)	0.09 (0.06–0.4)	2.9 (1.7–5.3)**	50.0 (16.2–366)**	18.9 (7.2–49.2)**
W-EBC (nmol/L)	< 0.5	< 0.5	1.1 (0.5–4.9)	25.6 (15.2–76.1)
W-U (μmol/mol creatinine)	< 0.06 (< 0.06 – 1.5)	< 0.06 (< 0.06 – 1.0)	1.2 (0.6–4.9)	8.2 (3.2–16.1)
MDA-EBC (nmol/L)	7.6 (7.0–8.5)	11.5 (6.3–14.6)	14.2 (12.4–16.6)**	26.5 (6.5–44.0)*

Co, 1 nmol/L = 58.9 ng/L, 1 μmol/mol creatinine = 0.52 μg/g creatinine; W, 1 nmol/L = 185 ng/L, 1 μmol/mol creatinine = 1.64 μg/g creatinine.

* *p* < 0.05 versus controls (Mann-Whitney test).

** *p* < 0.01.
